# Pushing the boundaries in autologous breast reconstruction: innovations from imaging to artificial intelligence

**DOI:** 10.3389/fsurg.2025.1679524

**Published:** 2025-09-26

**Authors:** Isabell Scherrer, Raymund E. Horch, Dominik Promny, Theresa Promny, Elisabeth Eschenbacher, Andreas Arkudas

**Affiliations:** Department of Plastic and Hand Surgery and Laboratory for Tissue Engineering and Regenerative Medicine, University Hospital Erlangen, Erlangen, Germany

**Keywords:** breast cancer, breast reconstruction, autologous breast reconstruction, 3D imaging, artificial intelligence

## Abstract

Breast cancer remains the most commonly diagnosed malignancy among women worldwide, with surgical intervention, ranging from breast-conserving procedures to total mastectomy, representing a cornerstone of curative treatment. In this context, breast reconstruction has become an essential component of comprehensive cancer treatment, addressing not only physical restoration but also playing a vital role in psychosocial rehabilitation and body image. Among the various reconstructive options, autologous tissue transfer has emerged as the preferred method for many patients, offering durable and natural-feeling results. In particular, abdominal-based free flaps such as the Deep Inferior Epigastric Perforator (DIEP) flap and the muscle-sparing Transverse Rectus Abdominis Myocutaneous (ms-TRAM) flap offer excellent results with reduced donor side morbidity. As the global number of breast cancer continues to rise, the demand for safe, individualized, and functionally superior reconstructive options rises as well. This article aims to provide a general overview of current surgical approaches and to highlight perspectives for future innovations in improving autologous breast reconstruction and patient satisfaction.

## From observation to precision: the evolution of preoperative imaging in autologous breast reconstruction

Since the pioneering days of free flap surgery, the approach to autologous breast reconstruction has undergone remarkable transformation (1, 2). Due to advanced wound care tools, wound bed preparation has made it possible to transplant free flaps to the chest in contaminated and irradiated areas at an earlier time point (3). The seminal introduction of his first “free abdominoplasty flap”, which basically was a free Transverse Rectus Abdominis Myocutaneous (TRAM) flap by Holmström (4), followed by the refinement of perforator flap techniques such as the Deep Inferior Epigastric Perforator (DIEP) flap introduced by Koshima (5), marked milestones that revolutionized reconstructive options after mastectomy. In the early phases of these techniques, flap planning relied heavily on tactile surgical experience and clinical acumen—primarily through direct observation of skin perfusion and rudimentary Doppler ultrasound to locate suitable perforators (6, 7). However, with growing recognition of the complexity of abdominal vascular anatomy and the critical importance of optimizing flap perfusion while minimizing donor site morbidity, a paradigm shift occurred (8, 9). The field has steadily moved toward increasingly sophisticated and standardized preoperative imaging protocols (10).

**Figure 1:**
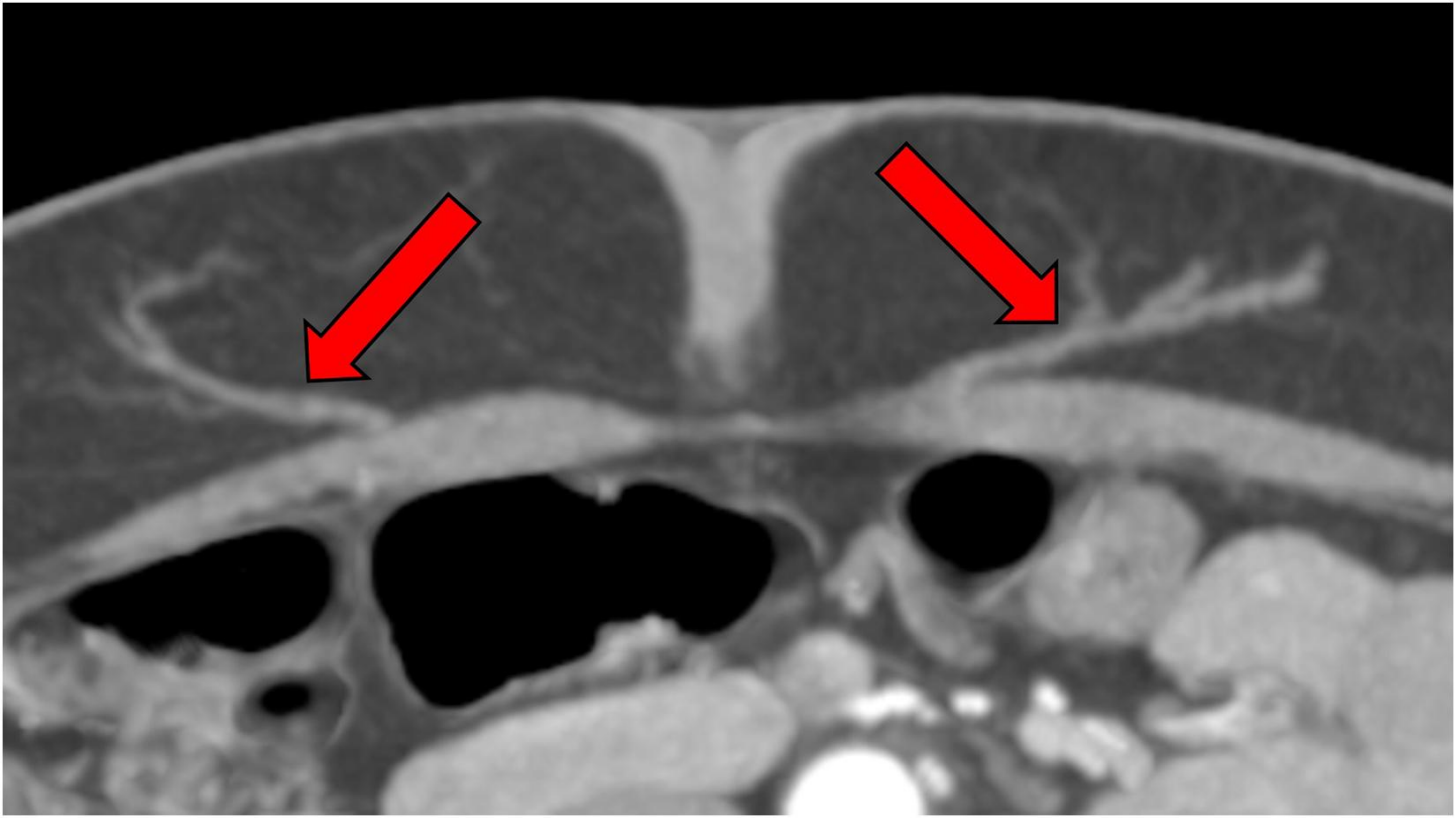
CTA-based perforator mapping enabling precise identification of vessel course and caliber, which facilitates tailored flap design and minimizes intraoperative dissection time.

Today, Computed Tomography Angiography (CTA) has emerged as the clinical gold standard for preoperative mapping of the perforators of the inferior epigastric artery (11) (see Figure 1). With near-perfect sensitivity, CTA offers unparalleled spatial resolution and precise anatomical visualization, dramatically improving surgical planning and intraoperative confidence (12). It represents a major leap forward from earlier tools such as handheld Doppler and Duplex sonography, which—though still valuable—lack the depth and clarity required for consistently reliable results in complex cases. Cinematic rendering of data acquired by CT-angiography allows for completely three-dimensional depicting of vessels and could well become an integrated part of the imaging algorithm (13, 14).

Magnetic Resonance Angiography (MRA) has become a valuable alternative for patients with contraindications to iodinated contrast or ionizing radiation, such as those with renal impairment or contrast allergies. While more resource-intensive and not yet universally available, MRA provides high-quality vascular imaging and continues to gain ground as an adjunct or substitute in selected scenarios (15). This method is promising but needs further validation.

Beyond vascular imaging, the field is rapidly embracing next-generation visualization tools. Techniques such as cinematic rendering and the creation of 3D-printed models of individual patients' vascular anatomy offer surgeons a tangible, spatially accurate reference to guide dissection and flap elevation (16, 17). These innovations are not only enhancing operative planning but also serve as powerful tools in surgical training and patient education (18). Furthermore modern Artificial Intelligence (AI) driven modalities are rapidly being developed and optimized for clinical application (19). The field of tissue engineering (TE) and regenerative medicine (RM) is also using 3D printing techniques with the aim to once produce tissue repair from the laboratory without the hitherto unavoidable donor site for autologous tissue, but those have not yet entered the clinical stage (20). Potential side effects of cultured cells in such replacement tissue need further investigations (21). Further on the effect of pre- or postoperative irradiation and appropriate imaging or influencing the radiation effects will be another step to further advance breast reconstruction (22).

Although only a limited number of studies have investigated the application of dynamic infrared thermography (DIRT) in DIEP reconstruction exists, it has been proposed that use of DIRT during the operation could allow the tailoring of the surgery and postoperative use may potentially identify vascularization problems in an early stage (23). Nevertheless, up to date additional high-quality studies are needed to ensure the true value for the pre-, per- and postoperative phase of DIEP-flap reconstructions.

**Figure 2:**
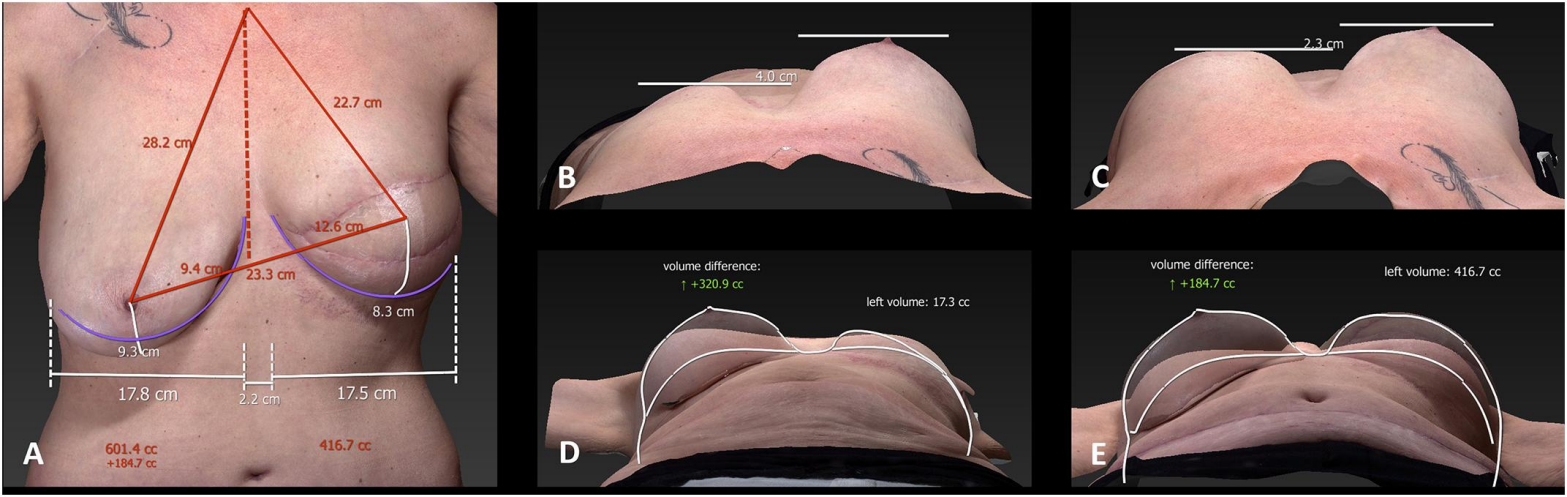
3D surface imaging of a patient after mastectomy of the left breast and autologous breast reconstruction. This kind of imaging is used for preoperative planning, providing volumetric assessment and symmetry simulation to improve patient–surgeon communication before breast reduction on the right breast. The panels **(A–E)** show the different measurements of the breasts. With these measurements the volume difference is calculated.

Three-dimensional surface imaging and volumetric simulation technologies have also entered clinical practice, providing a non-invasive means of assessing body contour, estimating flap volume, and simulating postoperative outcomes (9). These tools foster clearer communication between patient and surgeon and support shared decision-making by setting realistic expectations (24) (see Figure 2).

During surgery, real-time assessment of flap vascularity has been significantly advanced by the use of fluorescence angiography with indocyanine green (ICG) (25). This technique provides immediate visual feedback on tissue perfusion, enabling surgeons to identify poorly perfused areas early and adjust the surgical plan accordingly (26). As a result, the risk of partial flap necrosis is substantially reduced, contributing to improved surgical outcomes and flap viability.

## Advancements in surgical techniques and robotic integration

The landscape of reconstructive breast surgery is rapidly advancing toward less invasive, precision-driven methods, with a growing emphasis on robotic and microsurgical innovations. Robotic-assisted procedures, particularly with platforms like the Da Vinci Surgical System, have introduced the possibility of laparoscopic flap harvest with the aim to possibly reduce the length of the anterior rectus sheath fascial incision. It has also been speculated that this evolution could hold the promise of minimizing donor site morbidity and postoperative recovery times. However, these approaches often require transperitoneal access, which brings inherent risks such as bowel injury, adhesion formation, or postoperative ileus—factors that necessitate careful patient selection and surgical planning.

However, it needs to be mentioned that the robotic DIEP harvest technique involves entering the peritoneal cavity, unlike standard extraperitoneal techniques that preserve peritoneal integrity. This carries intraabdominal complications and potential risks of bowel injury, adhesions, seroma, especially in patients with prior surgeries (27). In these patients, adhesions may hinder robotic maneuverability and safe port placement. In addition, laparoscopy can lead to a loss of the peritoneal barrier and may increase postoperative discomfort and complications (28). The risk of multiple 8 mm–10 mm fascial defects by the port punctures needs to be taken into account together with the posterior rectus sheath violation by the intentional incision of the posterior rectus sheath (28). This may weaken abdominal wall integrity and should be considered. Robotic DIEP-flap harvest from the lower abdomen has been demonstrated to increase both ischemia time and total operative time. When considered in conjunction with the multiple port incisions, the claim that the technique is truly “minimally invasive” is called into question, despite the proposed reduction in fascial defects. Although the robotic-assisted DIEP flap technique therefore represents a notable advancement in microsurgical innovation, it is important to critically assess its limitations to avoid an overly favorable portrayal that may overlook significant surgical and logistical complexities. Future research should incorporate rigorous scientific evaluation, including data on hernia rates at both the fascial incision and robotic port sites, precise measurements of flap ischemia time (16, 29), and thorough cost–benefit analyses that address clinical outcomes, operative efficiency, and healthcare resource utilization.

In summary initial clinical data are encouraging, yet widespread adoption is tempered by longer operative times and the technical demands of setup and intraoperative coordination. The current state of development in robotic microsurgery needs to be further improved to definitely enter the daily clinical routine. While Wessel et al. suggest that the complete replacement of surgeons by robotic systems remains improbable, these technologies are expected to assume an increasingly impactful role in supporting and improving surgical performance. Continued technological advancement will necessitate rigorous research and well-designed clinical trials to optimize robotic platforms and substantiate their broader integration into routine surgical care (30).

A promising and increasingly studied approach in reconstructive breast microsurgery is vascularized lymph node transfer (VLNT), particularly when using abdominally based free flaps, as a therapeutic option for postmastectomy lymphedema (31). This method seeks to reestablish normal lymphatic drainage by transplanting viable lymphatic tissue along with its blood supply. Almadani demonstrated that simultaneous VLNT can be safely integrated with autologous breast reconstruction to treat or prevent breast cancer-related lymphedema. Nonetheless, they emphasize the need for further research to standardize protocols for data collection and to effectively report patient outcomes related to both lymphedema and immediate lymphatic reconstruction (32).

Preliminary patient-reported outcomes have been encouraging—many individuals report decreased reliance on compression garments, improved limb comfort, and a subjective sense of enhanced quality of life. However, when scrutinized under objective clinical parameters—such as limb circumference, bioimpedance, or volumetric reductions—the results from larger cohort studies have been modest and somewhat inconsistent.

These findings highlight the need for standardized outcome measures, better-defined surgical protocols, and improved patient selection criteria. Further prospective, controlled studies will be essential to clarify which patients are most likely to benefit, and under what clinical circumstances VLNT offers the most durable and clinically meaningful improvements in lymphatic function (33, 34).

## Postoperative monitoring

Conventional flap monitoring involves clinical evaluation of a skin island (35). Non-invasive and reliable methods for early identification of postoperative complications of free flaps that allow higher rates of salvage rate and reduce the need for specific staff with continuous on-site presence for flap monitoring have been investigated and proposed ever since free flap surgery became a clinical routine procedure (36). Lindelauf et al. reported that tissue oximetry following DIEP flap breast reconstruction can potentially facilitate a decrease in hospital costs since its readings enable physicians to intervene in an early stage of tissue malperfusion, contributing to minimizing complications and that it may eliminate the need for specialized postoperative care (37). However, based on the current literature, no firm conclusions can yet be drawn regarding cost-effectiveness of standard implementation.

While novel technologies such as surface probes (38), implantable Doppler probes (39), and flow couplers represent promising advancements in the intraoperative and postoperative monitoring of free flap anastomotic patency (40), their clinical implementation is still accompanied by important limitations and areas of uncertainty. These tools are designed to offer alternatives to traditional external skin paddles for monitoring, aiming to enhance early detection of complications, particularly venous insufficiency, without compromising the aesthetic outcome. For example, implantable Doppler probes provide continuous auditory signals indicating flow at the anastomotic site, while flow couplers integrate a Doppler sensor into the venous coupler ring, potentially allowing for non-invasive assessment of venous outflow. While surface and implantable monitoring systems hold substantial promise for improving flap surveillance, their clinical utility remains partially validated. Until larger, prospective, and ideally randomized studies confirm their efficacy and cost-effectiveness, these technologies should be considered as adjuncts, not replacements, to clinical judgment and traditional monitoring protocols.

Another innovation is the O2C (Oxygen to See) system, which noninvasively measures tissue oxygenation, hemoglobin concentration, and blood flow, offering real-time perfusion diagnostics to distinguish between arterial and venous complications (41, 42). Also, hyperspectral imaging alone or together with thermography has been propagated as another promising tool for perfusion controls in DIEP flaps (43, 44), similar to other application in reconstructive and hand surgery (45, 46). However, at the moment a lack of standardization hampers a more widespread clinical use and solid prospective studies are warranted.

## Artificial intelligence in reconstructive breast surgery

The integration of AI is rapidly advancing within the field of reconstructive surgery, offering transformative potential across the entire perioperative continuum. AI-driven tools are being developed and validated for a range of applications, from preoperative planning to intraoperative guidance and postoperative monitoring. Ozmen and coauthors developed a machine learning model to predict 30-day readmission risk using a large national surgical quality database. They reported a stacked machine learning approach that demonstrates a strong predictive capability for post-DIEP flap readmissions, with high sensitivity for identifying at-risk patients. The model's performance suggests clinical utility in preoperative risk stratification and resource allocation (47). Implementation could enable targeted intervention strategies to potentially reduce readmission rates in high-risk populations.

In the preoperative phase, machine learning algorithms trained on radiologic datasets -particularly from CTA and MRI scans—have demonstrated the ability to identify and rank perforators, thereby streamlining perforator flap planning and reducing both time and interobserver variability. These tools may enhance the precision of DIEP and ms-TRAM flap surgeries, optimizing donor site selection and potentially improving outcomes.

AI-based predictive modeling is also being explored to forecast patient-specific risk profiles (48, 49). By incorporating multivariate data such as body mass index (BMI), comorbidities, surgical technique, smoking behavior, and prior interventions, these models may assist surgeons in personalizing risk stratification, surgical decision-making, and patient counseling.

Intraoperatively, AI holds promise for real-time decision support. Applications under investigation include aesthetic outcome prediction, augmented surgical navigation, and integration with robotic platforms for automated or semi-automated tissue dissection. Combined with augmented reality (AR), AI could enable surgeons to visualize subsurface vascular anatomy or highlight critical structures dynamically, enhancing operative precision and safety (50, 51).

In the postoperative setting, AI-based platforms are being tested for automated flap monitoring. By analyzing serial photographs captured via smartphone or tablet, these systems could potentially detect early signs of vascular compromise or wound complications and alert the clinical team. While these approaches are promising—especially for outpatient follow-up or remote care—they rely on the availability of high-quality training datasets and must be critically evaluated for algorithmic bias, particularly those arising from sociodemographic, ethnic, or geographic disparities.

While emerging technologies in imaging, robotics, and AI hold significant promise, their current clinical integration is limited by heterogeneous study designs, small sample sizes, and short follow-up periods. High costs, restricted availability, and the need for specialized training also constrain widespread adoption. Furthermore, many AI tools have not undergone robust external validation, and their performance in diverse patient populations remains unclear. Future research should prioritize large-scale, multi-center trials, standardized outcome measures, and cost-effectiveness analyses to ensure these innovations can be safely and equitably implemented.

Additionally one obstacle to clinical implementation of the latest AI-driven technology is the question of lega liablity in case complications occur which harm the patient. This issue needs to be solved in the future to allow the introduction of AI technology into routine clinical practice. Evidence base for certain innovations is therefore at the moment naturally restricted to small series or early feasibility studies.

In summary, AI stands to redefine many aspects of reconstructive surgery by enhancing precision, efficiency, and personalization. However, its integration must be approached with methodological rigor, robust validation, and ethical consideration to ensure equitable and safe implementation in clinical practice.

## Conclusion

Autologous breast reconstruction stands at the forefront of innovation in reconstructive surgery, driven by rapid advancements in preoperative imaging, microsurgical techniques, robotic assistance, and the integration of artificial intelligence. The evolution from basic clinical assessment to high-resolution vascular imaging and dynamic intraoperative visualization has dramatically refined surgical planning and execution. Simultaneously, the emergence of robotics and microsurgical platforms enables greater precision, reduced invasiveness, and the potential for decreased donor-site morbidity.

Moreover, AI-powered tools offer new dimensions in personalized risk assessment, aesthetic outcome prediction, and automated postoperative monitoring, marking a paradigm shift toward data-driven, patient-specific surgical strategies. Collectively, these technological breakthroughs aim to improve surgical safety, reproducibility, and functional and aesthetic outcomes, while enhancing patient satisfaction and quality of life.

Nevertheless, the clinical integration of these innovations must proceed with methodological rigor and ethical oversight. Large-scale, multi-center trials are necessary to validate emerging techniques and technologies to ensure their equitable applicability across diverse patient populations. Challenges such as cost-effectiveness, training requirements, regulatory approval, and algorithmic transparency must be addressed proactively to facilitate responsible and sustainable implementation.

In summary, while autologous breast reconstruction has already made significant strides, it continues to evolve as a dynamic field at the intersection of surgical artistry and technological innovation. The path forward lies in harmonizing these advances with evidence-based practice and patient-centered care, thereby expanding access to safe, individualized, and high-quality reconstructive solutions.

## Data Availability

The original contributions presented in the study are included in the article/Supplementary Material, further inquiries can be directed to the corresponding author.
